# Social isolation and loneliness among Chinese older adults: Examining aging attitudes as mediators and moderators

**DOI:** 10.3389/fpsyg.2022.1043921

**Published:** 2022-12-06

**Authors:** Juanjuan Sun, Weikang Jiang, Haohao Li

**Affiliations:** ^1^School of Sociology and Population Studies, Renmin University of China, Beijing, China; ^2^Center for Population and Development Studies, Renmin University of China, Beijing, China; ^3^School of Public Administration, Xiangtan University, Xiangtan, China

**Keywords:** positive aging attitudes, negative aging attitudes, social isolation, loneliness, Chinese older adults

## Abstract

Due to labor migration and social changes, the Chinese elderly are facing significant social isolation, along with changes in aging attitudes. However, whether social isolation affects loneliness among the Chinese elderly and whether this relationship is moderated and mediated by aging attitudes is unclear. This empirical study aimed to respond to the above questions in the Chinese context, Based on the data from the 2014 China Longitudinal Aging Social Survey (N = 6,645), the results showed that social isolation is a positive predictor of loneliness; aging attitudes mediate the relationship between social isolation and loneliness. Social isolation affects the loneliness of the elderly partially by weakening positive aging attitudes and strengthening negative aging attitudes; aging attitudes moderate the effect of social isolation on loneliness. For those older adults with higher positive aging attitudes, social isolation has a much smaller effect on loneliness. While for those older adults with higher negative aging attitudes, social isolation has a more substantial effect on their feelings of loneliness. Our results indicate that less social isolation is an effective way to relieve loneliness, and maintaining higher positive aging attitudes and lower negative aging attitudes, is important for the Chinese elderly to prevent loneliness when facing social isolation.

## Introduction

Loneliness is one of the most prevalent psychological problems among older adults. The percentage of older adults reporting loneliness ranges from 20 to 40% in western countries (Ong et al., 2016). In China, about one-third of older adults feel lonely (Luo and Waite, 2014). Since the outbreak of COVID-19, many countries have imposed lockdown measures, making more people fall into loneliness (Odone et al., 2020). Loneliness causes a variety of problems. Empirical studies have shown that loneliness is highly associated with cognitive decline, depression, immune dysfunction, and other problems (Cacioppo and Hawkley, 2009; Cornwell and Waite, 2009; Park et al., 2014). In addition, loneliness increases the likelihood of mortality in older people (Holt-Lunstad et al., 2015). Similar findings have been found in China. Loneliness increases the risk of dementia, depression, and mortality risk among the Chinese elderly (Luo and Waite, 2014; Zhou et al., 2018; Bao et al., 2021).

Many empirical studies have shown that social isolation and loneliness are closely related (Alcaraz et al., 2019). In the past decades, aging and social transition have been taking place simultaneously in China, which makes a large number of the elderly in China face unprecedented social isolation. In 2020, China has more than 264 million people aged 65 and above, accounting for 18.70% of the total population (National Bureau of Statistics, 2020). Simultaneously, urbanization has accelerated the miniaturization of families, by 2020, the proportion of the elderly living alone and with their spouses reached 21.38 and 23.45%, respectively, (Xu et al., 2014; National Bureau of Statistics, 2020). Unlike western countries, Chinese culture is rooted in Confucian values, Chinese older adults are highly dependent on family members, especially on their children (Zheng and Chen, 2020), thus these changes may result in reduced social contact for older adults (Luo and Waite, 2014). Coupled with the fact that social networks of the elderly are intrinsically more likely to be in a reduced state due to stressful events may experience in old age, such as widowhood, retirement, and so on (Bao et al., 2021), aging people in China are at high risk of social isolation. It is worth exploring whether the increased social isolation of the elderly in China has also led to more loneliness.

Understanding how social isolation affects loneliness and whether there are ways to mitigate this impact is of great significance in protecting the mental health of the elderly. A few relevant studies have been conducted currently, for example. Li and Tang (2022) discovered that solitary activity significantly moderated the adverse effects of the low frequency of social contact with family members on loneliness among older Americans during the COVID-19 pandemic. Belvederi Murri et al. (2019) have found that physical exercise can not only improve the physical health of depressed patients, but also effectively combat depressive symptoms. However, all of the above studies have only focused on the role of external resources and ignored the role of older people’s subjective initiatives such as aging attitudes.

Aging attitudes refer to an individual’s beliefs, opinions, and expectations regarding the aging of oneself and others, and are usually divided into two dimensions: positive aging attitudes (PAA) and negative aging attitudes (NAA; Laidlaw et al., 2007). Aging attitudes are regarded as a psychological resource for the elderly (Korkmaz Aslan et al., 2019). According to stereotype embodiment theory, the surrounding culture shapes individuals’ attitudes toward aging, which in turn affects their functioning and health (Levy, 2009). Based on this theory, social isolation as a life experience may shape the aging attitudes of older people and thus affect their sense of loneliness. Existing studies have also found that self-perceptions of aging could mediate the relationship between social isolation and poor mental health outcomes (Bellingtier and Neupert, 2018). Besides, when faced with stressful events, positive attitudes toward aging play a protective role for older adults, while negative attitudes toward aging, in contrast, lead to more unsatisfactory reactions (Levy, 2009). Based on the above analysis, aging attitudes, as the intrinsic psychological resources of the elderly, are likely to play an important role in the relationship between social isolation and loneliness, however, there is still a lack of relevant research on the Chinese elderly.

Based on the above background, the primary objective of this study was to examine what role aging attitudes play in the relationship between social isolation and loneliness, whether they were mediators, moderators, or both. The secondary objective of this study was to examine whether social isolation was a positive predictor of loneliness in the Chinese context, which was also a prerequisite for examining the role of aging attitudes.

## Theoretical framework and hypothesis

### Theoretical framework

Social support theory suggests that social relationships and social networks can protect individuals’ physical and mental health through direct and buffering effects. Thus, a lack of social relationships and social support is one of the main causes of loneliness. According to this theory, older adults who are in social isolation are easier to feel lonely (Bareket-Bojmel et al., 2021). Stereotype embodiment theory suggests that the environment in which older adults live shapes their attitudes toward aging, which in turn affects their functioning and health (Levy, 2009). Based on the idea of stereotype embodiment theory, an older individual in social isolation whose attitudes towards aging are more likely to be different from those who are not in the isolated state, and thus his psychological feelings would be different accordingly than other groups.

**Figure 1:**
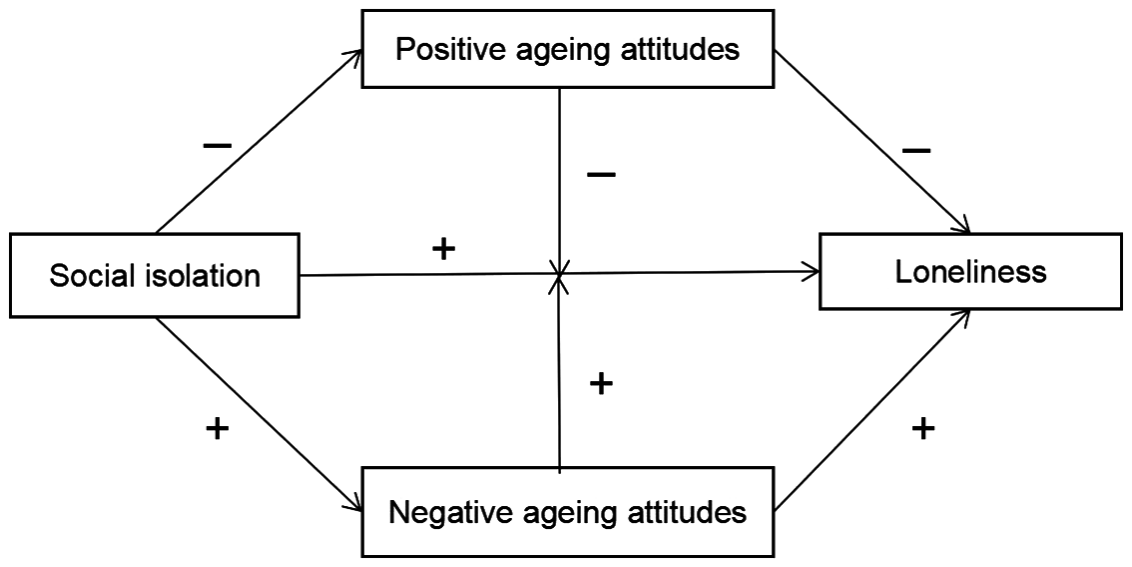
Research model.

Meanwhile, as a psychological resource, positive aging attitudes can play a buffering role when older adults face stress, and the opposite is true if they have negative aging attitudes (Mock and Eibach, 2011; Yamada et al., 2015). Based on the above theoretical analysis, the study structure of this paper is shown in Figure 1.

### Social isolation and loneliness

Social isolation represents an objective state in which the individual has little contact with society, resulting in a state of lack of social relations (Lubben et al., 2006; Wu and Sheng, 2020). The social networks of an individual usually comprise both family and friend networks (Shankar et al., 2011). For instance, a person living alone or in a rural area with less physical interaction with the populated world is socially isolated comparatively. In comparison, loneliness is more subjective and is typically defined as a feeling of isolation, a lack of social belonging, and an unpleasant experience of intimacy (Griffin, 2010). Loneliness has also been conceptualized as the sense of distress resulting from the discrepancy between an individual’s desired and actual social relationships (Peplau and Perlman, 1982).

A large body of research suggests that social isolation and loneliness are closely related. Social isolation is a strong predictor of loneliness, and socially isolated people often feel more lonely (Petersen et al., 2016; Tomova et al., 2020; Cheng et al., 2021). Some studies suggest, however, that loneliness is influenced by differences in psychological expectations and realistic relationships or by the quality of social interaction (Hawkley et al., 2008). In this case, social isolation does not necessarily cause loneliness, and lonely people are not necessarily in a state of social isolation (Coyle and Dugan, 2012). However, the association between social isolation and loneliness among older adults has been relatively little discussed in the Chinese context. Based on the above literature review, the first pair of competing hypotheses are proposed as:

*H1a*: There is a positive relationship between social isolation and loneliness among the Chinese elderly.

*H1b*: Social isolation has no effect on loneliness among the Chinese elderly.

### The mediating role of aging attitudes

Previous research has found that senior citizens with a positive aging attitudes report higher levels of mental health and better quality of life (Low et al., 2013; Bedaso and Han, 2021). In contrast, a negative concept of aging can result in physically frail and lower subjective well-being (Brown et al., 2016; Gale and Cooper, 2018).

Among the various contributing variables of aging attitudes, social relationships are considered to be the critical factor influencing aging attitudes (Laidlaw et al., 2007; Diehl et al., 2014). According to Diehl and Wahl (2010), one of the core factors of adults’ subjective aging experiences was the interpersonal interactions in their lives. In an empirical study, Rashid et al. (2014) discovered that older Malaysians with high levels of social support have more positive aging attitudes. One study found that social isolation and poor relationship quality can result in lower scores of aging perceptions (Santini et al., 2019). Another study found that friend isolation significantly affects older adults’ attitudes toward aging (Cheng et al., 2021). However, fewer studies point to differences in the effects of social isolation on different dimensions of older adults’ attitudes toward aging. That is, it remains to be proven whether social isolation weakens the positive aging attitudes and enhances the negative aging attitudes among Chinese older adults.

The way an individual views their aging process may cause significant changes in their social behavior and lead to loneliness. Some scholars believed that attitudes toward aging affect loneliness through self-fulfilling prophecies (Hu and Li, 2022; Segel-Karpas et al., 2022). In other words, older adults who subscribe to negative age stereotypes are more prone to experience feelings of loneliness.

Numerous studies in western countries have discovered substantial links between attitudes toward aging and older individuals’ loneliness. For example, based on three-waved surveys spanned 8 years, Hu and Li (2022) concluded that older persons with negative self-perceptions of aging are more likely to experience high levels of loneliness. Losada-Baltar et al. (2022) discovered that negative self-perceptions of aging, particularly self-perception as a burden, are associated with increased loneliness. Empirical studies examining the association between attitudes toward aging and loneliness among the Chinese elderly are scarce. According to Chen et al. (2021), both positive and negative attitudes toward aging significantly impact loneliness among the Chinese elderly, but there are considerable disparities between groups. Furthermore, Zeng et al. (2022) proposed that attitudes toward aging play an essential role in alleviating the loneliness of the elderly. As a result, we can conclude that attitudes toward aging significantly affect loneliness in older adults and that positive and negative aging attitudes may play diverse roles.

Through literature research, we found only one study that examined the relationship between attitudes toward aging, social connectedness, and loneliness (Hu and Li, 2022). Hu and Li’s study did not, however, address the potential mediating roles that aging attitudes may play. In other studies, Cheng et al. (2021) confirmed that aging attitudes mediate the relationship between social isolation and depression symptoms. In other words, social isolation may be an antecedent variable influencing attitude toward aging. In addition, Zeng et al. (2022) discovered that attitudes toward aging mediate the connection between activity patterns and loneliness in older adults. However, it remains unclear if the attitudes toward aging by the Chinese elderly has a mediating role between social isolation and loneliness. Based on the works mentioned, we propose H2a and H2b:

*H2a*: Aging attitudes by Chinese older adults may mediate the links between social isolation and loneliness.

*H2b*: Aging attitudes do not play a mediating role in the relationship between social isolation and loneliness among Chinese older adults.

### The moderator role of aging attitude

Based on previous studies, researchers often view aging attitudes as psychological resources, which can serve as a buffering role when suffering from adverse life events. In other words, having a positive self-perception of aging may be seen as a tool that helps shield the elderly from the harmful effects of adverse life events.

Among numerous studies, the moderating effect of attitudes toward aging remains controversial. Some studies have concluded that positive attitudes toward aging can mitigate the detrimental impacts of stressful events, while others have found that attitudes toward aging have no moderating effect. For example, Liu D. et al. (2020) found that the interaction between social support and negative aging attitudes significantly affects depression among older Chinese adults. Spitzer et al. (2019) suggested that older adults’ perception of aging can moderate the relationship between close social relationships and loneliness. These results revealed that aging attitudes may reduce the impact of isolation on loneliness. However, some studies have found no moderating effect of aging attitudes. One study suggested that aging attitudes did not moderate the relationship between the number of diagnosed mental health conditions and anxiety (Seow et al., 2022). Another survey about older Chinese people suggested that aging attitudes did not affect the connection between life satisfaction and perceived health (Liu J. et al., 2020). There are two main reasons for this: first, the inconsistencies in measurement instruments may lead to different results. Second, possible cultural differences in attitudes toward aging among older adults. For instance, a study indicated that older adults in societies with collectivism and individualism had different perspectives on aging perceptions (Brothers et al., 2017). Given these contradictory findings, the moderating role of aging attitudes between social isolation and loneliness should be explored further. We propose H3a and H3b:

*H3a*: Aging attitudes moderate the relationship between social isolation and loneliness.

*H3b*: Aging attitudes do not affect the relationship between social isolation and loneliness.

## Materials and methods

### Survey data and sample

The data for this study were obtained from the 2014 China Longitudinal Aging Social Survey (CLASS), which is a nationwide, continuous and large-scale social survey project by the Institute of Gerontology, Renmin University of China. 2014CLASS adopted a stratified multi-stage probability sampling method, selecting county-level areas as primary sampling units and village/neighborhood committees as secondary sampling units, and interviewing one elderly person per household. The 2014CLASS survey collected demographic, socio-economic, and family information of older people aged 60 and above in China. This survey ran from June 1 to August 31, 2014, covering 28 provinces, autonomous regions, and municipalities directly under the central government in China. The final sample covered 134 counties, districts, and 462 villages.

Among total of 11,511 samples in 2014 CLASS, 6645 samples have been included in this analysis with excluding the invalid samples and samples with existing missing values or errors related to this study.

### Measurement

#### Dependent variable

Loneliness was measured by three items from the Short Loneliness Scale (Hughes et al., 2004). Participants were asked, “How often do you feel you lack companionship?” “How often do you feel left out?” and “How often do you feel isolated from others?.” It was a three-point Likert scale, with higher scores associated with greater loneliness. In this study, Cronbach’s α was 0.708.

#### Explanatory variables

Explanatory variables in this study have been divided into independent variable-Social Isolation (SL) and intermediary variable-Aging Attitudes (AA). AA were measured by two dimensions: PAA and NAA.

#### Social isolation

Lubben Social Network Scale (LSNS-6) was used to measure the social isolation status of the elderly (Lubben et al., 2006). The 6-items in LSNS-6 represent family and friend networks. In terms of the family network, the respondent was asked how many relatives he/she: (a) sees or hears at least once a month? (b) could call for help? and (c) can talk about private matters? Each item ranged from 0 for “none” to 5 for “nine or more.”

The friend network is measured with the same questions as the family network. Thus, the total score of LSNS-6 ranged from 0 to 30, with a higher score representing a larger social network. In this study, the Cronbach’s α of family and friend networks were 0.739 and 0.846, respectively. According to the studies by Lubben et al., a total score of less than 12 is considered to be social isolation. This scale has been widely used in China and has good reliability and validity (Lubben et al., 2006; Wu and Sheng, 2020).

#### Aging attitudes

Aging attitudes were measured by the Attitudes to aging Questionnaire (AAQ), which has been proven to be a scale with high reliability and validity to measure the aging attitudes of older adults (Laidlaw et al., 2010). There are two dimensions of aging attitudes: PAA and NAA. PAA were measured by three items, including (a) The older people are, the better they are at dealing with life’s problems; (b) Wisdom grows with age; (c) There are many enjoyable things about growing old. It was a five-point Likert scale. The score of PAA ranges from 3 to 15, with higher scores representing more positive aging attitudes.

NAA was measured by four items. Respondent was asked (a) I feel old; (b) To me, aging is a process of continuous loss; (c) With growing old, it is more difficult for me to make new friends; and (d) I feel excluded due to my age. The score of NAA ranges from 4 to 20, a higher score means a greater degree of negative attitudes toward aging. In this study, Cronbach’s α were 0.598 for PAA and 0.691 for NAA.

#### Control variables

Some socio-demographic variables are controlled in our research, including age (in years), gender (0 = male, 1 = female), residential type (0 = urban, 1 = rural)，marital status (0 = married, 1 = unpartnered), education (0 = lower than middle school,1 = middle school and above), annual personal income (logged), ADL (activities of daily living with 6 items) and Chronic illness(0 = no chronic disease,1 = with chronic disease).

### Analytical strategies

The data were analysed using the statistical software package SPSS 25. The empirical analysis of this study consists of three procedures. The first step was to examine how social isolation affects older respondents’ loneliness by linear regression analysis, controlling for socio-demographic characteristics variables. Second, to test whether the PAA and NAA mediate the effect of social isolation on older adults’ loneliness, we used SPSS PROCESS macro (model4), implementing the bootstrapping method, which was iterated 5,000 times and had 95% bias-corrected confidence intervals (Hayes, 2013).

In the third step, multilevel linear regression models were used to test whether the moderating effect of PAA and NAA on the relationship between social isolation and loneliness was significant. In Model I, the control variables and independent variable are included; In Model II and Model III, the interaction term of PAA and social isolation, and NAA and social isolation was added, respectively, based on Model I. To further explore the moderating effect, we plotted the moderating effect of PAA and NAA by adding or subtracting the standard deviation of the moderating variables and exploring the effect of social isolation on loneliness at different levels of the moderating variables.

## Results

### Descriptive statistics

Table 1 presents the socio-demographic characteristics of the elders and statistics of all variables. The average age of the respondents was 68.92 years. 55.53% were male, 65.93% lived in rural areas, 71.78% had spouses, and 53.89% were educated below the middle school. The average annual income(logged) of the respondents was 9.36 (SD = 1.31), the mean score of activity of daily living (ADL) was 6.13 (SD = 0.79), 71.70% had chronic diseases, and nearly one-third (30.26%) were in social isolation. The average loneliness score was 3.66 (SD = 1.31), the average positive aging attitude scale score was 8.33 (SD = 3.04), and the average negative aging attitude scale score was 12.81 (SD = 3.95).

**Table 1:** Descriptive statistics.

Variables	Mean	SD	Min	Max
**Age**	68.92	7.33	60	97
**Gender**				
Female (n, %)	2,955	44.47		
Male (n, %)	3,690	55.53		
**Residence**				
Urban (n, %)	2,264	34.07		
Rural (n, %)	4,381	65.93		
**Marriage**				
Married (n, %)	4,770	71.78		
Unpartnered (n, %)	1875	28.22		
**Education**				
Lower than middle school (n, %)	3,581	53.89		
Middle school and above (n, %)	3,064	46.11		
**Income (logged)**	9.36	1.31	4.09	13.77
**ADL**	6.13	0.79	6	18
**Chronic illness**				
No chronic disease (n, %)	1,418	27.30		
with chronic disease (n, %)	4,831	71.70		
**Social isolation**				
In social isolation (n, %)	2011	30.26		
Not in social isolation (n, %)	4,634	69.74		
**Loneliness**	3.66	1.31	3	9
**Positive aging attitudes**	8.33	3.04	3	15
**Negative aging attitudes**	12.81	3.95	4	20

N = 6,645, SD, standard deviation.

The correlations of the core variables in this study are shown in Table 2. Social isolation had a significant positive correlation with loneliness (*r* = 0.136, *p* < 0.001) and negative aging attitudes (*r* = 0.177, *p* < 0.001), and a significant negative correlation with positive aging attitudes (*r* = −0.100, *p* < 0.001). There was a significant negative correlation between positive aging attitudes and negative aging attitudes (*r* = −0.210, *p* < 0.001). Positive aging attitudes were significantly negatively correlated with loneliness (*r* = −0.145, *p* < 0.001), while negative aging attitudes were significantly positively correlated with loneliness (*r* = 0.298, *p* < 0.001).

**Table 2:** Correlations of core variables.

	1	2	3	4
**1.Social isolation**	–			
**2.Positive aging attitudes**	−0.100***	**–**		
**3.Negative aging attitudes**	0.177***	−0.210***	–	
**4.Loneliness**	0.136***	−0.145***	0.298***	–

N = 6,645, *p < 0.05; **p < 0.01; ***p < 0.001.

### Hypothesis testing

#### Analysis of main and mediating effects

**Figure 2:**
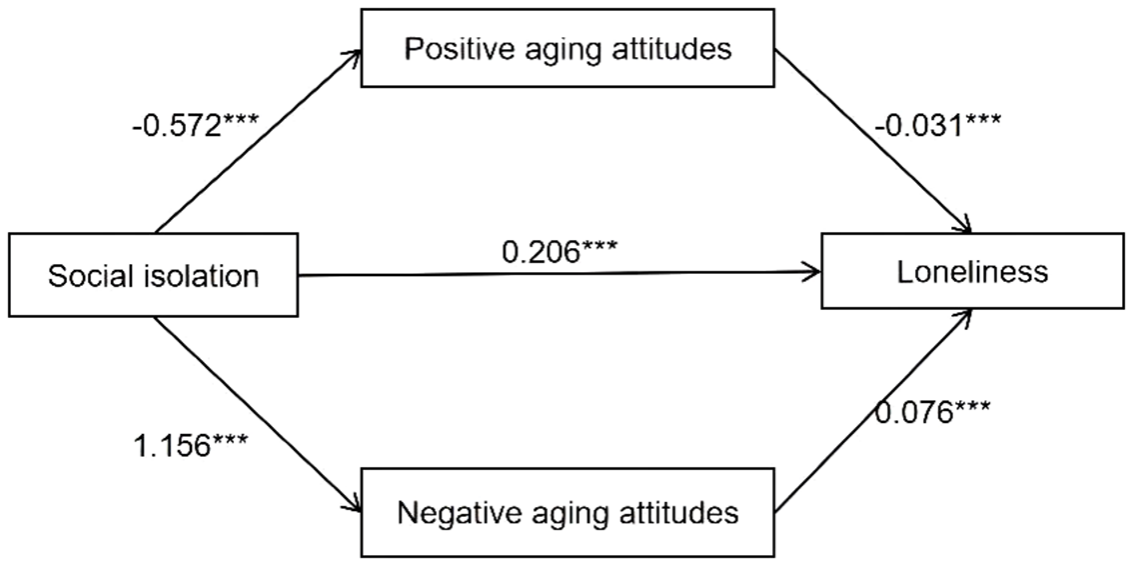
The mediating effect of aging attitudes on social isolation and loneliness. *p < 0.05; **p < 0.01; ***p < 0.001.

To investigate the effect of social isolation on loneliness among Chinese older adults and whether positive aging attitudes and negative aging attitudes play a mediating role, we used SPSS25.0 software and Process Model 4 (Hayes, 2013). The results are shown in Figure 2.

Social isolation significantly affects older adults’ loneliness (B = 0.206, SE = 0.033,t = 16.452, **p** < 0.001, LLCI = 3.422, ULCI = 4.347). That is, older adults who are socially isolated have higher loneliness levels than those who are not isolated. H1a is verified. Social isolation negatively influenced PAA (B = −0.572, SE = 0.081, *t* = −7.071, *p* < 0.001, LLCI = −0.731,ULCI = −0.413), and PAA reduce loneliness (B = 0.031, SE = 0.005, *t* = −6.227, *p* < 0.001, LLCI = −0.041, ULCI = −0.021); The result shows social isolation would strengthen NAA(B = 0.206, SE = 0.033, *t* = 16.452, *p* < 0.001, LLCI = 3.422, ULCI = 4.347) and NAA might further reinforce loneliness in older adults (B = 1.156, SE = 0.098, *t* = 11.775, *p* < 0.001, LLCI = 0.964, ULCI = 1.349).

Table 3 shows that there are three pathways through which social isolation affects loneliness. In addition to its direct effects, social isolation also indirectly increases loneliness through two mediating pathways-positive aging attitudes (B = 0.018, SE = 0.014, LLCI = 0.011, ULCI = 0.026) and negative aging attitudes (B = 0.088, SE = 0.009, LLCI = 0.071, ULCI = 0.107), H2a is supported.

**Table 3:** Test results of mediating effect analysis.

Model pathways	B	SE	95% CI	
			Lower	Upper
**Direct effects**				
SI → LO	0.206***	0.033	0.142	0.271
**Indirect effects**				
SI → PAA → LO	0.018***	0.004	0.011	0.026
SI → NAA → LO	0.088***	0.009	0.071	0.107
Total effect	0.106***	0.010	0.087	0.127

SI, social isolation; PAA, positive aging attitude; NAA, negative aging attitude; LO, loneliness;

*p < 0.05; **p < 0.01; ***p < 0.001.

#### Analysis of the moderating effect

To explore the role of buffering of aging attitudes, we use hierarchical regression to analyze whether PAA and NAA moderate the effect of social isolation on loneliness. The results in Table 4 reveal that the interaction between social isolation and PAA had a significant inhibitory effect on loneliness (Model2, B = −0.154, SE = 0.011, LLCI = −0.071, ULCI = −0.029), while the interaction between social isolation and NAA had a significant facilitative effect on loneliness (Model3, B = 0.320, SE = 0.008, LLCI = 0.046, ULCI = 0.79).

**Table 4:** A hierarchical regression analysis for moderating effects in the relationship between social isolation and loneliness.

Variables	Model 1		Model 2		Model 3	
	B	SE	B	SE	B	SE
Age	−0.037**	(0.002)	−0.037**	(0.002)	−0.056***	(0.002)
Gender	−0.019	(0.033)	−0.020	(0.032)	−0.011	(0.032)
Urban	0.012	(0.039)	0.008	(0.039)	−0.014	(0.038)
Marriage	0.206***	(0.037)	0.205***	(0.037)	0.201***	(0.036)
Education	−0.007	(0.036)	−0.008	(0.036)	0.012	(0.035)
Income	−0.129***	(0.015)	−0.124***	(0.015)	−0.093***	(0.014)
ADL	0.052***	(0.020)	0.044***	(0.019)	0.037**	(0.019)
Chronic illness	0.084***	(0.035)	0.075***	(0.035)	0.049***	(0.034)
SI	0.110***	(0.034)	0.243***	(0.093)	−0.217***	(0.116)
PAA			−0.071***	(0.006)		
PAA × SI			−0.154***	(0.011)		
NAA					0.186***	(0.005)
NAA × SI					0.320***	(0.008)
Constant	4.375***	(0.233)	4.692***	(0.238)	3.728***	(0.231)
Observations	6,645		6,645		6,645	
R-squared	0.096		0.111		0.154	

SI, social isolation; PAA, positive aging attitude; NAA, negative aging attitude; LO, loneliness.

*p < 0.05; **p < 0.01; ***p < 0.001.

**Figure 3:**
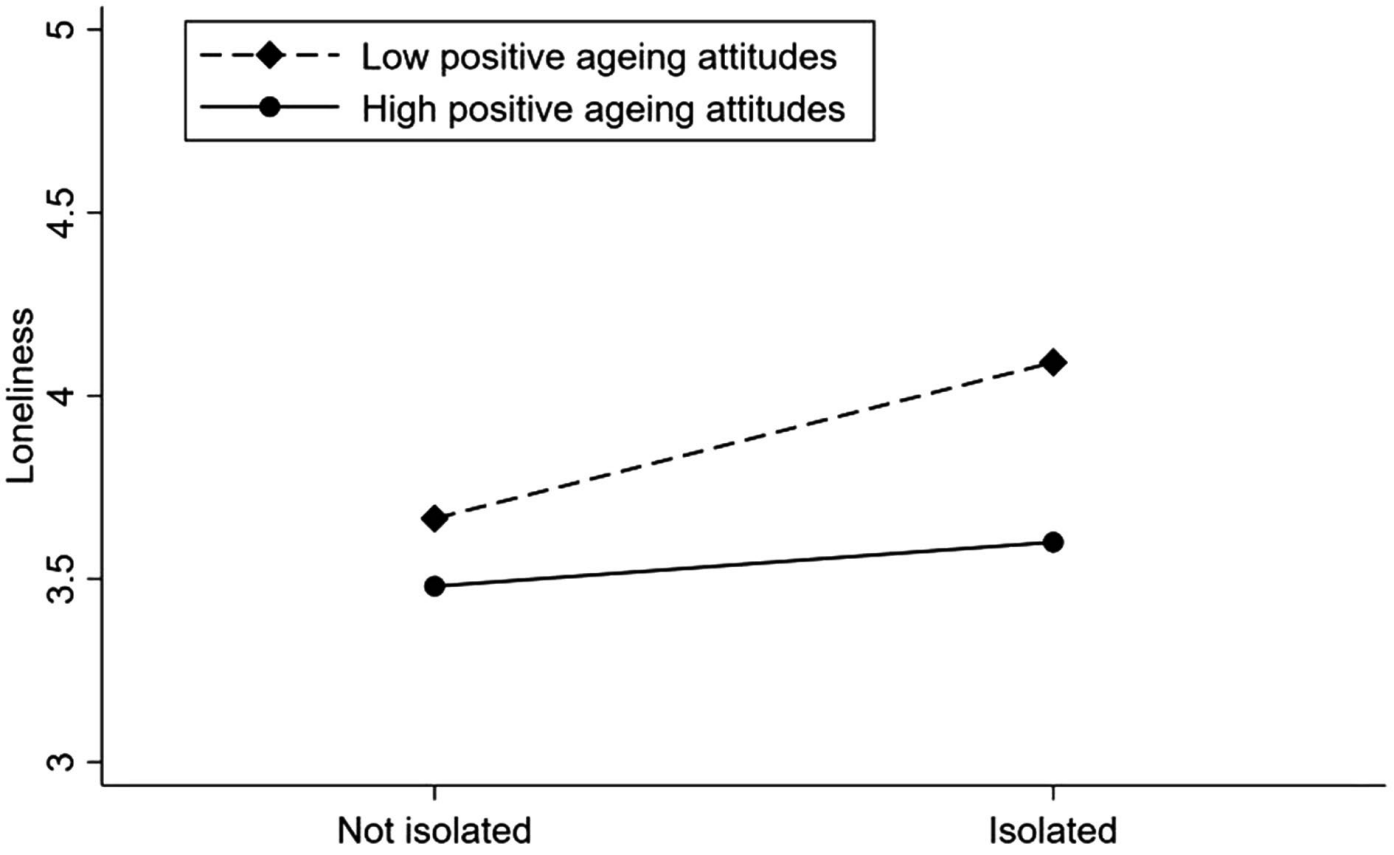
Loneliness among older adults who were not socially isolated or isolated with different levels of positive aging attitudes.

**Figure 4:**
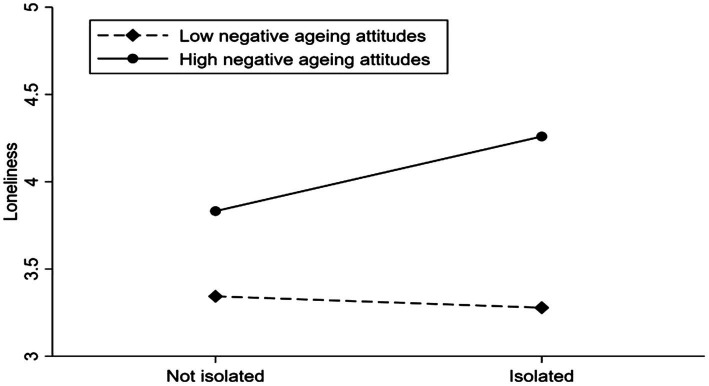
Loneliness among older adults who were not socially isolated or isolated with different levels of negative aging attitudes.

We constructed the moderating effect maps to further analyze the moderating effects of PAA and NAA. Figure 3 shows that PAA moderated the positive association between social isolation and loneliness. The positive correlation will be weaker when older adults are at high levels of PAA, while Figure 4 shows that NAA moderates the positive association between social isolation and loneliness, and that the positive association will be stronger when older adults are at higher levels of NAA.

## Discussion

In line with previous studies (Cheng et al., 2021; Stockwell et al., 2020), older Chinese adults who are socially isolated feel far more lonely. Thus, our study provides direct evidence indicating that the Chinese elderly would suffer from loneliness due to lack of social interaction with family members and friends.

Our results verifies the relationship between aging attitudes and loneliness among the Chinese elderly, which has been found in prior research (Chen et al., 2021). On the one hand, older adults with a positive attitude toward aging may experience less loneliness. On the other hand, negative attitudes toward aging would increase their sense of loneliness. According to the Stereotype embodiment theory (Levy, 2009), loneliness is closely related to the perception of age during aging. Our research demonstrates that attitudes toward aging, both positive and negative, have a significant impact on loneliness in older adults. In addition, negative aging attitudes have a stronger effect on loneliness than positive aging attitudes. So, fighting against the effects of negative aging attitudes is crucial. Unlike previous research that employed mixed methods with a small sample (Kuru Alici and Kalanlar, 2021), this study used large, representative survey data from China, the world’s largest elderly population, confirming the significant relationship between aging attitudes and loneliness.

Consistent with earlier research (Li et al., 2021), our study points to the mediation role of attitude toward aging in the relationship between social isolation and loneliness. Firstly, social isolation can weaken older adults’ positive attitudes toward aging, which in turn affects their sense of loneliness. Therefore, the mediating role of positive aging attitudes becomes essential for alleviating loneliness among older adults. Secondly, the higher level of loneliness among older adults who are socially isolated can be attributed to the fact that these seniors have negative attitudes toward aging, which strengthens the sense of loneliness. This demonstrated that negative attitudes toward aging play a mediating role (Brown et al., 2016) and further diminish the mental health of older adults (Low et al., 2013). In other words, older adults who suffer from social isolation may have lower positive aging attitudes and higher negative aging attitudes, thus, further contributing to a strong sense of loneliness. Contrary to previous studies showing that only negative aging attitude acts as the mediator (Moor et al., 2006; Li et al., 2021), our study confirmed two mediating pathways of aging attitudes between social isolation and loneliness. This gives us more detailed results that help us figure out how these variables are connected in the Chinese context.

In addition to demonstrating the role of attitudes toward aging as a mediator, this study reveals that positive attitudes toward aging may mitigate the negative impacts of social isolation on loneliness among the Chinese elderly. In contrast, negative attitudes toward aging would exacerbate the detrimental effects of social isolation on loneliness. More specifically, when socially isolated, older persons with positive aging attitudes may respond more proactively, which lowers the sense of loneliness. Many studies attribute the moderate role of positive aging attitudes to the fact that they can be seen as a psychological resource (Jang et al., 2006; Mock and Eibach, 2011), thus, playing a protective role in preventing the further spread of the detrimental consequences of social isolation. Contrarily, older adults with negative attitudes towards aging tend to be more susceptible to impairment, which adds to a stronger sense of loneliness. This result is similar to past findings that suggest that negative aging attitudes play a moderator role and further increase negative consequences among older adults (Choi et al., 2019). However, this differs from previous study concluded that positive aging attitudes have no moderating effect (Li et al., 2021). The present study verified the moderating effects of both positive and negative aging attitudes.

Our research has important implications for future professional interventions and policy initiatives. First, the important role of subjective attitudes on social isolation and loneliness emphasizes that improving the positive attitudes towards aging is meaningful to decrease the level of loneliness of the elderly caused by social isolation, especially during covid-19. Cognitive programs while implimentating active aging strategy for those isolated older adults should be emphasized. For instance, by fostering an age-friendly environment and acknowledging the role of older adults, Chinese society might reduce negative attitudes toward aging, At the same time, the social participation of older adults can be encouraged to enhance positive self-identity, and improving the quality of intergenerational relations has also been found to be an effective way to improve the positive aging attitude of older Chinese (Liu et al., 2022). Second, for family members and social workers of older adults, future intervention and treatment programs for loneliness may include assessing and measuring aging attitudes. In addition, strengthening social networks, such as family and friend networks, will help maintain psychological resources and improve positive attitude towards aging. Third, considering the profound negative impact of social isolation on loneliness, social policy needs to pay more attention to the social isolation of older adults during covid-19, especially for those living alone and in empty nests, to enhance their contact and interaction as a way to reduce loneliness. Finally, few interventions and resources are available for Chinese isolated older adults. Setting up a system to take care of isolated older adults may be essential to avoid loneliness.

## Limitation

We have some limitations. First, this study used cross-sectional data for validation, presenting a correlation rather than a causal relationship. To better understand the causal relationships between social isolation, aging attitudes, and loneliness among Chinese older adults, longitudinal data are needed. In addition, the effects of different dimensions of social isolation on loneliness among older adults need further research. Second, during the covid-19 pandemic and lockdown, an increasing number of older adults are in social isolation and loneliness, which we have not discussed due to data limitations. In the future, we will focus on related topics. Last, although our study investigated the mediating and moderating role of aging attitudes, the mechanism underlying the association between social isolation and loneliness was still not fully understood. More factors in the relationship between social isolation and loneliness in older adults need to be discussed.

## Data availability statement

Publicly available datasets were analyzed in this study. This data can be found at: http://class.ruc.edu.cn.

## Ethics statement

Ethical review and approval were not required for the study on human participants in accordance with the local legislation and institutional requirements. The patients/participants provided their written informed consent to participate in this study.

## Author contributions

WJ and HL collected the data and performed the data analyses. JS, WJ and HL drafted and revised the manuscript. All authors contributed to the article and approved the submitted version.

## Funding

This research was supported by National Social Science Foundation Project “Support System of the Function of Family-based Elderly Care in the National Strategy of Actively Coping with Population Aging” (21ASH014) and the School of Interdisciplinary Studies, Renmin University of China.

## Conflict of interest

The authors declare that the research was conducted in the absence of any commercial or financial relationships that could be construed as a potential conflict of interest.

## Publisher’s note

All claims expressed in this article are solely those of the authors and do not necessarily represent those of their affiliated organizations, or those of the publisher, the editors and the reviewers. Any product that may be evaluated in this article, or claim that may be made by its manufacturer, is not guaranteed or endorsed by the publisher.
